# Solvent-Dependent GC–MS Fingerprinting of Lipophilic Constituents in *Syzygium polyanthum* Leaves: A Baseline Study for Future Greener Extraction Optimization

**DOI:** 10.3390/molecules31111932

**Published:** 2026-06-03

**Authors:** Frangky Jessy Paat, Sanriomi Sintaro

**Affiliations:** 1 Agrotechnology Study Program, Bioengineering, Faculty of Agriculture, Sam Ratulangi University, Manado 95115, Indonesia; 2 Information System Study Program, Faculty of Mathematics and Natural Science, Universitas Sam Ratulangi, Jl. Kampus Unsrat, Manado 95115, Indonesia; sanriomi@unsrat.ac.id

**Keywords:** GC–MS, green extraction, lipophilic constituents, n-hexane fraction, *Syzygium polyanthum*

## Abstract

*Syzygium polyanthum* (Wight) Walp., commonly known as Indonesian bay leaf or *Daun salam*, is widely used as a culinary and traditional botanical resource. However, region-specific information on its lipophilic constituents remains limited, and the sustainability implications of solvent-dependent phytochemical profiling are rarely addressed. This study characterized the GC–MS-detectable volatile lipophilic constituents of a selected nonpolar fraction of *S. polyanthum* leaves collected from Paniki Bawah, Mapanget District, Manado, Indonesia, using GC–MS, while evaluating solvent-related limitations for future greener extraction strategies. Dried leaf powder was macerated with 96% ethanol, followed by liquid–liquid partitioning with n-hexane and ethyl acetate. The n-hexane fraction was separated by silica gel column chromatography, and a TLC-selected fraction was analyzed by GC–MS. Compound annotation was supported by NIST 17 library matching, retention-index comparison using a C8–C40 n-alkane series, diagnostic ion evaluation, solvent blank analysis, and triplicate injections. Ethanolic extraction of 900 g dried powder yielded 87.0 g crude extract (9.67%). The n-hexane, ethyl acetate, and aqueous fractions yielded 5.98%, 21.15%, and 72.87%, respectively. GC–MS analysis tentatively annotated 11 compounds representing 94.44% of the total normalized peak area. The major constituents were palmitic acid, phytol, squalene, and neophytadiene. All annotations showed match scores of 90–98%, ΔRI values of 4–8 units, and RSD values of 1.86–3.27%. Although ethanol use, sunlight drying, solvent recovery, and recirculating chiller-assisted evaporation partially aligned with green chemistry principles, the use of n-hexane and chloroform means that the workflow should not be described as fully green. This study provides a baseline GC–MS fingerprint to support future greener extraction optimization.

## 1. Introduction

The extraction of natural products is increasingly expected to satisfy not only phytochemical objectives, but also environmental, safety, and sustainability requirements. In green chemistry, extraction should be designed to reduce solvent consumption, energy demand, waste generation, and the use of hazardous petroleum-derived or chlorinated solvents wherever possible [1,2]. Solvent selection is therefore a central issue in phytochemical research, especially when plant extracts are intended for food, pharmaceutical, nutraceutical, or botanical-product applications. Water and ethanol are among the more acceptable solvents from a green chemistry perspective because of their lower toxicity, broader availability, and compatibility with many plant-derived materials [3,4]. Analytical greenness also requires attention to waste generation, operational safety, and the overall environmental burden of the procedure [5]. However, the extraction of lipophilic constituents remains challenging because many low-polarity metabolites are poorly recovered by water or aqueous ethanol alone.

Although green extraction principles encourage the replacement of hazardous solvents, conventional organic solvents are still frequently used in natural-product studies for fractionation, enrichment, and instrumental sample preparation. n-hexane can effectively enrich nonpolar constituents, but it is a petroleum-derived solvent with recognized safety and environmental concerns [3]. Chloroform is also problematic from a green chemistry perspective because it is a chlorinated solvent and is not preferred in sustainable analytical workflows. Therefore, studies involving n-hexane or chloroform should not be described as fully green extraction processes unless solvent reduction, solvent recovery, safer replacement, or comparative greenness assessment is clearly demonstrated. When such solvents are used, their methodological role and environmental limitations should be stated transparently.

*Syzygium polyanthum* (Wight) Walp., commonly known as Indonesian bay leaf or *Daun salam*, is an aromatic plant widely used in Indonesian cuisine and traditional health practices. Its frequent household use, broad availability, and long history of consumption make this species relevant for the development of safe, quality-controlled, and sustainable botanical products [6,7]. The genus *Syzygium* has been reviewed as a source of nutritionally and biologically relevant metabolites with reported antioxidant, anti-inflammatory, antimicrobial, and antihyperglycemic potential [7]. Specifically, *S. polyanthum* has attracted attention for its food-preservative potential and reported biological activities [8,9]. Its antibacterial and antidiabetic relevance has also been reported in previous studies [10,11,12,13]. However, plant chemical composition can vary according to geographic origin, harvest season, drying procedure, extraction solvent, fractionation method, and analytical conditions. Region-specific chemical profiling is therefore important for reproducibility, quality control, and future marker selection.

Previous phytochemical studies on *S. polyanthum* have largely focused on crude extracts, polar or semi-polar metabolites, biological activity, or application-oriented evaluation. For example, LC–MS-based work has provided useful information on polar and semi-polar constituents of *S. polyanthum* leaves [6], while bioactivity-guided and metabolomics-based studies have contributed to the understanding of antidiabetic and related pharmacological potential [12,13]. Syabana et al. also performed fractionation using solvents of different polarities and generated multiple fractions for biological and chemical evaluation. However, information on the lipophilic GC–MS-detectable volatile constituents of *S. polyanthum* leaves from clearly documented local sources in North Sulawesi remains limited. A region-specific nonpolar fingerprint can provide complementary information to previous polar and bioactivity-oriented studies and may serve as a reference for future extraction optimization and comparative quality-control studies.

GC–MS is a useful analytical platform for profiling volatile compounds in plant-derived nonpolar fractions. However, compound identification by GC–MS must be interpreted cautiously, particularly in complex botanical matrices. Library matching alone is insufficient for definitive structural confirmation because co-elution, overlapping mass spectra, and structurally similar compounds may lead to misannotation [14,15,16]. Therefore, GC–MS-based phytochemical studies require supporting information such as spectral match quality, retention-index comparison, diagnostic ions, blank analysis, replicate injection, and peak-area reproducibility [17]. Retention-index comparison is particularly useful in GC–MS because it provides chromatographic support for mass-spectral library matching and can improve compound-identification confidence [18]. When authentic standards are not used, detected compounds should be reported as tentatively annotated rather than definitively confirmed.

In the present study, ethanol was used as the primary extraction solvent for *S. polyanthum* leaves, followed by polarity-based partitioning and chromatographic handling to obtain a selected nonpolar fraction for GC–MS analysis. The use of ethanol, natural sunlight drying, solvent recovery, and recirculating chiller-assisted evaporation provided partial alignment with green chemistry principles. However, n-hexane was still used for nonpolar enrichment, and chloroform was used for GC–MS sample preparation. These steps represent clear methodological limitations. Therefore, the present work is positioned as a solvent-dependent baseline fingerprinting study rather than a fully optimized green extraction protocol.

Accordingly, this study aimed to characterize the volatile and semi-volatile lipophilic constituents of a selected nonpolar fraction of *S. polyanthum* leaves collected from Paniki Bawah, Mapanget District, Manado, North Sulawesi, Indonesia, using GC–MS. The specific objectives were: (i) to document the extraction and fractionation yields of a region-specific *S. polyanthum* sample; (ii) to tentatively annotate major lipophilic constituents using GC–MS supported by library matching, retention-index comparison, diagnostic ions, blank analysis, and replicate injection; (iii) to classify the detected compounds according to major chemical classes; and (iv) to evaluate the solvent-related limitations of the workflow in relation to future greener extraction strategies. By distinguishing baseline chemical fingerprinting from a fully green extraction process, this study provides a transparent foundation for future optimization using safer solvent systems, solvent-reduction strategies, and intensified green extraction techniques.

## 2. Materials and Methods

### 2.1. Study Design and Experimental Workflow

This study was designed as a solvent-dependent phytochemical fingerprinting study of the selected nonpolar fraction of *Syzygium polyanthum* leaves. The workflow consisted of plant material collection and authentication, drying and powder preparation, ethanolic extraction, liquid–liquid partitioning, silica gel column chromatography of the n-hexane fraction, TLC-guided fraction selection, and GC–MS-based chemical profiling.

The study was intended to generate a baseline lipophilic chemical fingerprint that can support future development of greener extraction strategies. Ethanol was selected as the primary extraction solvent because it is generally more acceptable for botanical, food-related, and pharmaceutical applications than many petroleum-derived or chlorinated solvents. However, n-hexane was used for nonpolar enrichment, and chloroform was used for GC–MS sample preparation. Therefore, these solvents were considered methodological limitations of the workflow.

**Figure 1:**
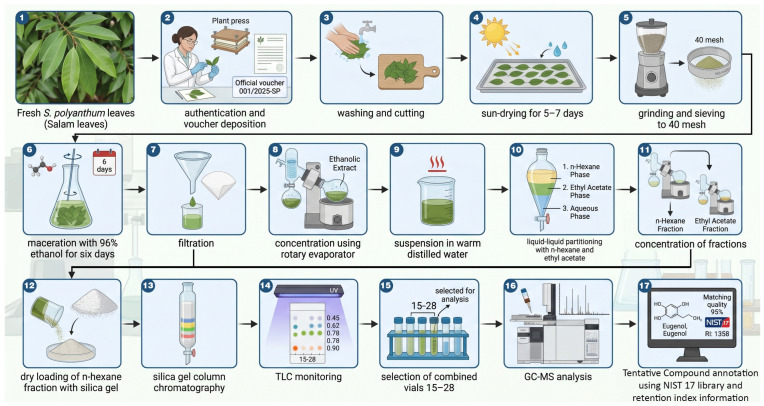
Workflow of extraction, fractionation, chromatographic handling, and GC–MS analysis of *Syzygium polyanthum* leaves.

The workflow was partially aligned with green chemistry principles through the use of ethanol as the primary extraction solvent, natural sunlight drying, solvent recovery, and recirculating chiller-assisted evaporation. Nevertheless, because n-hexane and chloroform were still used, the method was not considered a fully green extraction process. The overall experimental workflow, including plant material preparation, ethanolic extraction, liquid–liquid partitioning, chromatographic handling, TLC-guided fraction selection, and GC–MS analysis, is summarized in Figure 1.

### 2.2. Plant Material Collection and Authentication

Fresh leaves of *Syzygium polyanthum* (Wight) Walp. were collected in June 2023 during the dry season from Paniki Bawah, Mapanget District, Manado, North Sulawesi, Indonesia. The collection site was located at approximately 1°30′43″ N, 124°54′54″ E. The plant material was authenticated by the Department of Biology, Faculty of Mathematics and Natural Sciences, Universitas Sam Ratulangi, Manado. A voucher specimen was deposited at the institutional herbarium under voucher number SP-Mapanget-0623.

A total of 3000 g fresh leaves was collected. The leaves were washed with clean running water to remove dust and adhering impurities, separated from petioles, and cut into smaller pieces to improve drying uniformity. The plant material was dried under direct sunlight for 5–7 days at ambient temperature of approximately 28–32 °C until brittle. The dried leaves were ground and sieved to obtain 40-mesh dried leaf powder. The final dried powder weight used for extraction was 900 g. Before extraction, the powder was stored in an airtight container at room temperature, approximately 25 °C, and protected from light.

### 2.3. Chemicals, Reagents, and Instruments

The chemicals, reagents, and instruments used in this study are summarized in Table 1. Ethanol 96% was used as the primary extraction solvent. Distilled water was used to suspend the crude ethanol extract before partitioning. n-hexane and ethyl acetate were used for liquid–liquid partitioning and column elution, while chloroform was used as the solvent for GC–MS sample preparation. Silica gel 60 was used as the stationary phase for column chromatography and silica gel 60 GF254 was used for sample adsorption prior to column loading. A C8–C40 n-alkane standard mixture was used for retention-index calculation.

The solvents were selected according to their analytical roles in the extraction and fractionation workflow. Ethanol was used to obtain a broad crude extract, distilled water was used to disperse the crude extract before polarity-based partitioning, n-hexane was used to enrich nonpolar lipophilic constituents, ethyl acetate was used to recover semi-polar constituents, and chloroform was used to dissolve the selected nonpolar fraction before GC–MS injection.

From a green chemistry perspective, ethanol and water represented the more favorable components of the workflow, whereas n-hexane and chloroform represented methodological limitations. Therefore, the solvent system used in this study was considered a conventional solvent-dependent analytical workflow rather than a fully green extraction method.

The materials and instruments listed in Table 1 were selected to support polarity-based extraction, fractionation, and chemical profiling of *S. polyanthum* leaves. Ethanol was used as the primary extraction solvent to obtain a broad crude extract, while distilled water, n-hexane, and ethyl acetate were used to separate the extract into fractions with different polarity ranges. The n-hexane fraction was prioritized for GC–MS analysis because it was expected to contain GC–MS-detectable volatile lipophilic constituents. Although ethanol and water are more favorable from a green chemistry perspective, the use of n-hexane and chloroform represents a methodological limitation that should be addressed in future solvent-reduction or solvent-replacement studies.

### 2.4. Ethanolic Extraction by Maceration

Dried *S. polyanthum* leaf powder weighing 900 g was extracted using 96% ethanol by maceration. The powder was placed in a 5 L amber glass Erlenmeyer flask, and 3000 mL of ethanol was added per maceration cycle. The container was wrapped in aluminum foil to protect the extract from light. Maceration was performed at room temperature, approximately 27–30 °C, for six days. During extraction, the mixture was shaken three times daily.

The extraction was carried out in three cycles using a total ethanol volume of 9000 mL. After each cycle, the extract was filtered through Whatman No. 1 filter paper. The combined ethanolic filtrates were concentrated under reduced pressure using a rotary evaporator at 45 °C and 175 mbar to obtain a viscous crude ethanol extract. The final crude ethanol extract weighed 87.0 g, corresponding to an extraction yield of 9.67% based on the dried powder weight.

Maceration was selected because it is simple and suitable for preliminary phytochemical profiling. However, conventional maceration is solvent-intensive and time-consuming. These characteristics were considered limitations of the present extraction workflow [19].

### 2.5. Liquid–Liquid Partitioning

The crude ethanol extract weighing 87.0 g was suspended in 400 mL of distilled water warmed to 50 °C. The warm aqueous phase was used to facilitate dispersion of the crude extract and to provide a suitable medium for polarity-based partitioning. Liquid–liquid partitioning was first performed with n-hexane using 300 mL per partition cycle for three cycles, giving a total n-hexane volume of 900 mL for partitioning. The remaining aqueous phase was then partitioned with ethyl acetate using 300 mL per partition cycle for three cycles, giving a total ethyl acetate volume of 900 mL for partitioning.

After solvent removal, three fractions were obtained: n-hexane fraction, ethyl acetate fraction, and remaining aqueous fraction. The n-hexane fraction weighed 5.2 g, corresponding to 5.98% of the crude ethanol extract. The ethyl acetate fraction weighed 18.4 g, corresponding to 21.15%, while the aqueous fraction weighed 63.4 g, corresponding to 72.87%.

The n-hexane fraction was selected for further chromatographic handling and GC–MS analysis because it was expected to contain relatively nonpolar GC–MS-detectable volatile constituents, including fatty acid derivatives, hydrocarbons, terpenoid-related compounds, and other lipophilic metabolites suitable for GC–MS profiling [16,20]. Because n-hexane is a petroleum-derived solvent, this step should be regarded as a conventional analytical fractionation procedure rather than a green extraction step.

### 2.6. Column Chromatography and TLC Monitoring

The total n-hexane fraction weighing 5.2 g was mixed with 10 g of silica gel 60 GF254 until a free-flowing dry powder was obtained. This dry loading step was performed to improve sample handling and facilitate chromatographic separation. The dried sample mixture was loaded onto a silica gel column containing 150 g of silica gel 60 with a particle size of 0.063–0.200 mm. The column dimension was 3 cm × 60 cm.

Column chromatography was performed using a gradient elution system of n-hexane and ethyl acetate with increasing polarity from 100:0 to 0:100 *v*/*v*. Each gradient step used 250 mL of solvent mixture. Fractions were collected in 15 mL vials, and a total of 120 vials were obtained.

Fraction monitoring was performed using TLC with n-hexane:ethyl acetate 9:1 *v*/*v* as the developing solvent. TLC plates were visualized under UV light at 254 nm and 366 nm, followed by iodine vapor exposure. Based on TLC similarity and the presence of a dominant spot with an approximate Rf value of 0.65, vials 15–28 were combined as the selected nonpolar fraction for GC–MS analysis.

The TLC-guided selection of vials 15–28 was intended to reduce sample complexity before GC–MS analysis and to obtain a more interpretable chromatographic profile of the selected nonpolar constituents.

### 2.7. GC–MS Analysis

GC–MS analysis was performed at Narkobafopol, Forensic Chemistry Division, on the selected combined nonpolar fraction obtained from vials 15–28 after silica gel column chromatography. The selected fraction was dissolved in chloroform before injection.

GC–MS data were acquired using a Shimadzu GCMS-QP2010 Ultra system with GCMSsolution Ver. 4.45 as the acquisition software. Separation was performed using an HP-5MS capillary column, Agilent 19091S-433, with dimensions of 30 m × 0.25 mm × 0.25 μm. The injection volume was 1.0 μL, and the sample was injected in split mode with a split ratio of 1:50. The injector temperature was maintained at 250 °C. Helium with 99.999% purity was used as the carrier gas at a constant flow rate of 1.0 mL/min.

The oven temperature program started at 50 °C and was increased at a rate of 10 °C/min to 280 °C, followed by a final hold time of 10 min. The mass spectrometer was operated in electron ionization mode at 70 eV. The ion source temperature was set at 230 °C, and the interface temperature was set at 280 °C. Mass spectra were acquired over a scan range of *m*/*z* 35–500, with a solvent delay of 4.0 min.

A pure chloroform solvent blank was analyzed to monitor possible solvent-derived or system-derived peaks. The selected sample was injected in triplicate to evaluate chromatographic reproducibility. The C8–C40 n-alkane standard mixture was analyzed under the same chromatographic conditions for retention-index calculation. Because authentic reference standards were not used for final compound confirmation, the GC–MS results were interpreted as tentative compound annotations rather than definitive structural identifications.

The GC–MS conditions summarized in Table 2 were used to obtain a reproducible chromatographic and mass spectral profile of the selected nonpolar fraction. The HP-5MS column and electron impact ionization mode were suitable for profiling volatile compounds detectable under the applied GC–MS conditions in plant-derived nonpolar fractions. The use of triplicate injections, solvent blank analysis, NIST 17 library matching, and retention index calculation was intended to improve the reliability of compound annotation. Nevertheless, because authentic reference standards were not used, all detected compounds were reported as Tentatively annotated rather than definitively confirmed.

### 2.8. Compound Annotation and Retention Index Calculation

Compound annotation was performed by comparing the acquired mass spectra with reference spectra in the NIST 17 mass spectral library. Agilent MassHunter Unknowns Analysis was used for post-acquisition processing and library-search support. A minimum weighted match score of ≥85% was used as the initial criterion for tentative annotation.

Retention-index information was used to support compound annotation. Retention indices were calculated using a C8–C40 n-alkane standard series analyzed under the same chromatographic conditions. The calculated retention indices were compared with reference retention-index values reported in the NIST 17 library or relevant literature.

For each tentatively annotated compound, retention time, molecular formula, molecular weight, experimental retention index, reference retention index, retention-index difference, match score, diagnostic ions, normalized peak area, replicate variability, and identification status were recorded. Peaks detected in the solvent blank or showing poor reproducibility across replicate injections were not considered reliable sample-derived annotations.

Relative peak area percentages were calculated by normalization of total ion chromatogram peak areas. These values were used to describe relative GC–MS signal contributions under the applied analytical conditions. They were not treated as absolute compound concentrations because detector response, volatility, thermal stability, and ionization efficiency may differ among compounds.

Because authentic standards were not used to confirm the detected compounds, all compounds were reported as tentatively annotated. Definitive identification would require confirmation using authentic reference standards and, where necessary, additional orthogonal analytical methods [15,16,21].

### 2.9. Green Chemistry Data and Methodological Limitations

Green chemistry-related information was recorded to evaluate the environmental relevance and limitations of the workflow. The parameters included solvent type, solvent volume, extraction time, solvent recovery, drying method, evaporation condition, and the analytical role of each solvent.

The total ethanol volume used during maceration was 9000 mL. The total n-hexane volume used was 2275 mL, consisting of 900 mL for liquid–liquid partitioning and 1375 mL for column chromatography. The total ethyl acetate volume used was 2275 mL, consisting of 900 mL for liquid–liquid partitioning and 1375 mL for column chromatography. Chloroform was used only for analytical sample preparation, with a total volume of approximately 50 mL.

Ethanol and n-hexane were recovered using rotary evaporation, with an approximate solvent recovery rate of 75%. The extraction process also incorporated simple energy-saving or resource-saving steps, including natural sunlight drying and the use of a recirculating chiller during solvent evaporation. However, energy consumption and total waste generation were not quantitatively measured in this study.

The use of ethanol, sunlight drying, solvent recovery, and recirculating cooling provided partial alignment with green chemistry principles. Nevertheless, the continued use of n-hexane and chloroform limited the greenness of the overall method. Therefore, this workflow was regarded as a baseline solvent-dependent fingerprinting method rather than a fully green extraction protocol.

Table 3 summarizes the environmental relevance and limitations of each major step in the experimental workflow. The use of ethanol as the primary extraction solvent, natural sunlight drying, solvent recovery, and a recirculating chiller provided partial alignment with green chemistry principles. However, the workflow still involved n-hexane for nonpolar enrichment and chloroform for GC–MS sample preparation, both of which limit the overall greenness of the method. Therefore, the present study should be interpreted as a baseline chemical fingerprinting study rather than a fully optimized green extraction protocol. The information generated here can support future comparisons with greener extraction systems, such as ethanol-water mixtures, bio-based solvents, ultrasound-assisted extraction, or microwave-assisted extraction [4,19].

## 3. Results and Discussion

### 3.1. Extraction and Fractionation Yield

Ethanolic maceration of 900 g dried *Syzygium polyanthum* leaf powder produced 87.0 g of crude ethanol extract, corresponding to an extraction yield of 9.67%. The crude extract was then subjected to liquid–liquid partitioning using n-hexane and ethyl acetate, followed by recovery of the remaining aqueous fraction. The extraction and fractionation yields are summarized in Table 4.

The extraction and fractionation yields were obtained from one preparative extraction batch. The GC–MS peak-area data, in contrast, were evaluated from triplicate injections of the selected nonpolar fraction. This distinction is important because the yield values describe preparative recovery, whereas the GC–MS data describe analytical repeatability.

The use of ethanol as the primary extraction solvent was selected to obtain a broad crude extract while maintaining partial compatibility with green chemistry considerations. However, the subsequent use of n-hexane and ethyl acetate for liquid–liquid partitioning indicates that the overall process should be interpreted as a conventional solvent-dependent fractionation workflow rather than a fully green extraction process.

Table 4 shows that the aqueous fraction represented the largest proportion of the crude ethanol extract, indicating that most extracted constituents were polar or water-dispersible. In contrast, the n-hexane fraction showed the lowest yield, but this smaller fraction was chemically relevant because it was expected to enrich GC–MS-detectable volatile lipophilic constituents suitable for GC–MS analysis.

The low yield of the n-hexane fraction does not necessarily indicate low analytical importance. In polarity-based fractionation, a low-yield nonpolar fraction may still contain diagnostically useful lipophilic constituents, including fatty acids, terpenoid-related compounds, hydrocarbons, tocopherols, and phytosterols. Therefore, the n-hexane fraction was selected for further chromatographic handling and GC–MS profiling as a baseline nonpolar chemical fraction.

From a green chemistry perspective, the fractionation result also highlights a limitation of the present workflow. Although ethanol was used as the primary extraction solvent, n-hexane was still required for nonpolar enrichment. Therefore, future studies should compare this conventional n-hexane-based workflow with safer solvent systems or intensified extraction methods that can recover similar lipophilic constituents with reduced environmental and safety burden.

### 3.2. Selection of the Nonpolar Fraction for GC–MS Analysis

GC–MS analysis was performed on the selected nonpolar fraction obtained from combined vials 15–28 after silica gel column chromatography. This fraction was selected based on TLC monitoring, which showed a dominant spot with an approximate Rf value of 0.65 using n-hexane:ethyl acetate 9:1 *v*/*v* as the developing solvent.

The purpose of this TLC-guided fraction selection was to reduce the complexity of the n-hexane fraction before GC–MS analysis. Complex crude plant fractions may contain overlapping compounds that complicate chromatographic separation and mass spectral interpretation. Therefore, analysis of a selected combined fraction was considered more appropriate for obtaining an interpretable baseline lipophilic fingerprint.

However, this approach also means that the GC–MS profile reported in this study represents only the selected nonpolar fraction, not the complete chemical composition of the crude ethanol extract, the total n-hexane fraction, or the whole plant material. Therefore, the results should be interpreted as a method-specific and fraction-specific chemical fingerprint.

### 3.3. Tentative Annotation of Compounds by GC–MS

A total of 11 compounds were tentatively annotated from the selected nonpolar fraction based on GC–MS analysis. These compounds represented 94.44% of the total normalized peak area. The major tentatively annotated constituents were palmitic acid, phytol, squalene, and neophytadiene, with relative peak areas of 24.12 ± 0.48%, 18.67 ± 0.52%, 15.34 ± 0.38%, and 12.45 ± 0.31%, respectively.

The applied annotation criteria included NIST 17 library matching, retention-index comparison, diagnostic ion evaluation, solvent blank assessment, and triplicate injection. The match scores ranged from 90% to 98%, while the retention-index differences between experimental and reference values ranged from 4 to 8 units. The RSD values ranged from 1.86% to 3.27%, indicating acceptable repeatability across triplicate injections.

Despite this analytical support, all compounds were classified as tentative, RI-supported annotations rather than confirmed identifications because authentic reference standards were not used. The reported area percentages represent normalized GC–MS signal contributions under the applied analytical conditions and should not be interpreted as absolute compound concentrations.

The main GC–MS results are summarized in Table 5. Detailed annotation parameters, including molecular formula, molecular weight, experimental RI, reference RI, ΔRI, match score, diagnostic ions, and RSD values, are provided in Supplementary Table S1.

In Table 5, area percentages are presented as mean ± SD from triplicate injections. Detailed retention-index and spectral-matching parameters are provided in Supplementary Table S1. Compounds were classified as tentative, RI-supported annotations because authentic reference standards were not used.

The low ΔRI values of 4–8 units and high match scores of 90–98% indicate acceptable agreement between the acquired chromatographic behavior and library-based mass spectral annotation. The RSD values below 3.5% also support acceptable injection repeatability for the selected fraction. These parameters strengthen the reliability of the GC–MS fingerprint while maintaining an appropriate level of caution in compound identification.

**Figure 2:**
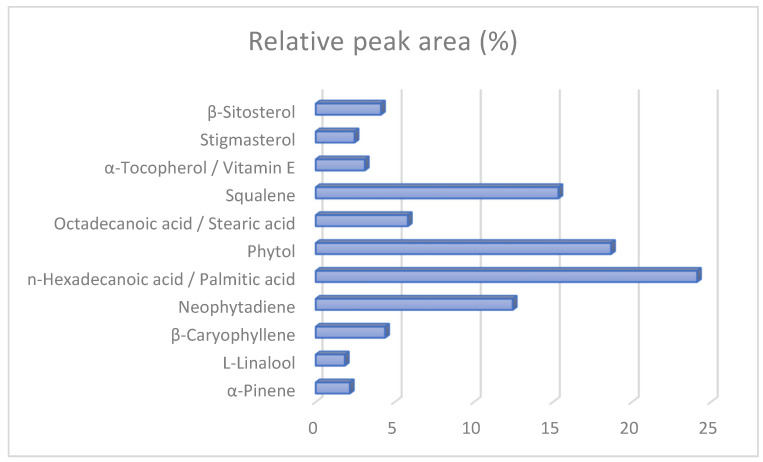
Relative peak area distribution of Tentatively annotated compounds in the selected nonpolar fraction of *Syzygium polyanthum* leaves.

Figure 2 shows the relative GC–MS signal distribution of the tentatively annotated compounds. Palmitic acid, phytol, squalene, and neophytadiene showed the largest normalized peak-area contributions, indicating that the selected nonpolar fraction was enriched in fatty acid and isoprenoid-related lipophilic constituents. Minor compounds, including α-pinene, L-linalool, β-caryophyllene, α-tocopherol, stigmasterol, and β-sitosterol, contributed smaller relative areas but remain chemically relevant as components of the selected lipophilic fraction.

### 3.4. Chemical-Class Distribution

To improve the interpretation of the GC–MS profile, the Tentatively annotated compounds were grouped into major chemical classes according to their structural features and functional groups. The chemical-class distribution is presented in Table 6.

Class-based interpretation is useful because it reduces the risk of overinterpreting individual compound assignments. In complex botanical fractions, individual compound annotation may be affected by co-elution, spectral similarity, and differences in detector response, whereas chemical-class patterns provide a more conservative overview of the fraction composition.

Table 6 indicates that the selected nonpolar fraction was dominated by diterpene-related compounds, fatty acids, and triterpene hydrocarbons. Diterpene-related compounds represented the largest combined area contribution, mainly due to phytol and neophytadiene, which together accounted for 31.12% of the annotated peak area. Fatty acids were also prominent, with palmitic acid and stearic acid contributing a combined area of 29.94%. Squalene represented 15.34% of the annotated profile, while phytosterols, tocopherols, and smaller volatile terpenoids contributed lower but chemically relevant proportions.

**Figure 3:**
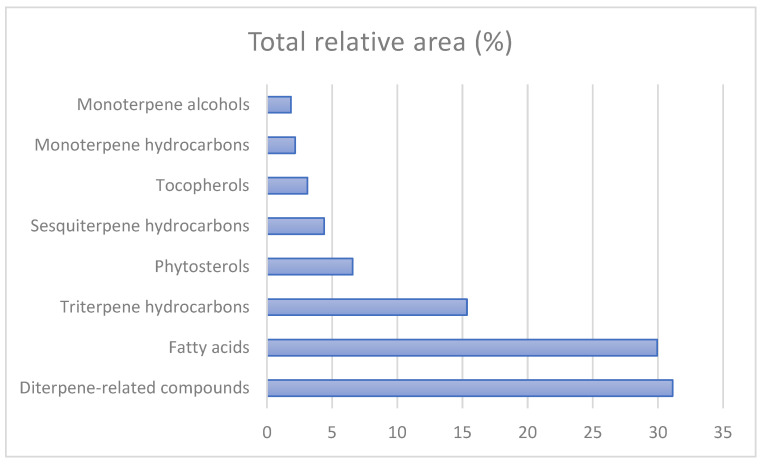
Chemical-class distribution of Tentatively annotated constituents in the selected nonpolar fraction of *Syzygium polyanthum* leaves.

Figure 3 provides a class-level overview of the selected nonpolar fraction. The dominance of diterpene-related compounds, fatty acids, and triterpene hydrocarbons supports the interpretation that the fractionation procedure enriched lipophilic plant constituents. This class-based pattern is more appropriate for interpreting a baseline GC–MS fingerprint than making strong biological claims for individual compounds.

### 3.5. Interpretation of Major Tentatively Annotated Constituents

Palmitic acid was the most abundant tentatively annotated compound, contributing 24.12 ± 0.48% of the total normalized peak area. Stearic acid was also detected at 5.82 ± 0.14%. These fatty acids are common lipid-derived constituents in plant extracts and are frequently detected in nonpolar or semi-nonpolar GC–MS profiles.

The detection of palmitic acid and stearic acid is consistent with the use of n-hexane fractionation and silica gel chromatographic enrichment, both of which favor relatively nonpolar constituents. However, the presence of these fatty acids should not be interpreted as evidence of biological activity in the present study because no direct bioactivity assay was performed on the selected fraction.

Phytol was the second major tentatively annotated constituent, with a relative peak area of 18.67 ± 0.52%. Neophytadiene was detected at 12.45 ± 0.31%. Together, phytol and neophytadiene accounted for 31.12% of the annotated peak area.

Phytol and neophytadiene are diterpene-related compounds that may be associated with chlorophyll-derived or isoprenoid-related plant constituents. Their detection is reasonable in a leaf-derived nonpolar fraction. Nevertheless, their functional relevance in *S. polyanthum* should be investigated through targeted quantification and biological or physicochemical assays rather than inferred only from GC–MS detection.

Squalene was detected at 15.34 ± 0.38%, making it one of the major tentatively annotated constituents of the selected fraction. Squalene is a highly lipophilic triterpene hydrocarbon, and its detection supports the interpretation that the selected fraction was enriched in nonpolar isoprenoid-related metabolites.

The presence of α-tocopherol, stigmasterol, and β-sitosterol further indicates that the selected fraction contained lipid-associated plant constituents. However, because authentic standards were not used, these assignments should remain tentative. Future work should confirm these compounds using authentic reference standards and targeted quantification.

The volatile terpenoid portion of the profile included α-pinene, L-linalool, and β-caryophyllene. These compounds contributed smaller relative areas than the fatty acids and higher-molecular-weight lipophilic compounds.

Although these volatile terpenoids were minor in terms of normalized peak area, their annotation was supported by spectral and retention-index agreement, as shown by match scores above 90%, ΔRI values of 4–5, and RSD values below 3%. These compounds are chemically relevant because *S. polyanthum* is an aromatic leaf material. Their presence may contribute to the volatile character of the selected fraction, but sensory, aroma, or bioactivity implications were not directly evaluated in this study.

### 3.6. Comparison with Previous Syzygium polyanthum Studies and Novelty of the Present Work

The present study complements previous phytochemical and biological studies of *S. polyanthum* by focusing specifically on a selected nonpolar fraction from leaves collected in Manado, North Sulawesi (Table 7). Previous reports have provided information on polar or semi-polar constituents, biological activity, food-preservative potential, antidiabetic relevance, antimicrobial properties, and other applications of *S. polyanthum*. In contrast, the current study provides a solvent-dependent GC–MS fingerprint of GC–MS-detectable volatile lipophilic constituents.

A previous study by Syabana et al. used a solvent-gradient fractionation approach involving n-hexane, acetone, and water, resulting in 42 fractions that were evaluated for antidiabetic-related activity and chemical profiling [12]. In contrast, the present work focuses on RI-supported GC–MS annotation of lipophilic constituents in a selected nonpolar fraction from a different geographic origin. This distinction addresses the need to clarify the unique contribution of the present work relative to previous fractionation studies.

The comparison indicates that the present study does not duplicate previous bioactivity-oriented work. Instead, it provides complementary information by focusing on a selected nonpolar fraction, applying GC–MS with retention-index support and replicate injection data, and explicitly discussing solvent-related limitations in relation to future greener extraction strategies. The current profile should therefore be interpreted as a region-specific, method-specific, and fraction-specific chemical fingerprint rather than a universal profile of *S. polyanthum* leaves.

This distinction is important for future standardization. A chemical fingerprint generated from a selected nonpolar fraction may be useful as a reference for comparison with future green extraction approaches, but it cannot replace comprehensive phytochemical profiling of the whole extract or direct quality-control validation using authenticated marker compounds.

### 3.7. Analytical Quality and Limitations of Compound Identification

The reliability of GC–MS-based compound annotation in this study was supported by NIST 17 library matching, retention-index comparison, diagnostic ion evaluation, solvent blank analysis, and triplicate injection. All tentatively annotated compounds showed match scores of 90–98% and ΔRI values of 4–8 units, indicating acceptable agreement between spectral and chromatographic evidence. In addition, RSD values of 1.86–3.27% indicated acceptable injection reproducibility.

Nevertheless, the compound assignments remain tentative because authentic reference standards were not used for final confirmation. Absolute quantification was also not performed. Therefore, the reported area percentages should be interpreted as relative GC–MS signal contributions rather than true concentrations. Future studies should confirm the major compounds using authentic standards and targeted calibration, especially if these compounds are proposed as chemical markers for quality control or green extraction optimization.

A major limitation of GC–MS-based botanical profiling is the possibility of compound misannotation when identification relies only on spectral library matching. The inclusion of retention-index comparison and diagnostic ions reduces this risk, but does not completely eliminate it. Therefore, the identification status in this study is reported as tentative, RI-supported rather than confirmed.

### 3.8. Green Chemistry Implications and Future Method Optimization

The present workflow partially aligns with green chemistry principles through the use of ethanol as the primary extraction solvent, natural sunlight drying, solvent recovery, and recirculating chiller-assisted evaporation. These features may reduce some environmental and operational burdens compared with workflows that rely entirely on petroleum-derived or chlorinated solvents.

However, the overall method cannot be described as a fully green extraction process because n-hexane was used for nonpolar enrichment and chloroform was used for GC–MS sample preparation. Both solvents represent important environmental and safety limitations. Therefore, the current study should be interpreted as a baseline solvent-dependent fingerprinting study rather than a completed green extraction method.

The main value of the present GC–MS profile is that it provides a reference point for future solvent-replacement and extraction-optimization studies. Future work should compare the current n-hexane-based nonpolar fractionation with safer solvent systems such as ethanol-water mixtures, bio-based solvents, or intensified green extraction techniques, including ultrasound-assisted extraction and microwave-assisted extraction.

Future green extraction studies should evaluate not only the number and relative abundance of detected compounds, but also extraction yield, solvent consumption, solvent recovery, waste generation, energy demand, extraction time, analytical performance, and safety profile. A comparative greenness assessment would be necessary before any future workflow can be claimed as a genuinely greener extraction method for *S. polyanthum* leaves.

## 4. Conclusions

This study provided a region-specific and solvent-dependent GC–MS fingerprint of the selected nonpolar fraction of *Syzygium polyanthum* leaves collected from Paniki Bawah, Mapanget District, Manado, North Sulawesi, Indonesia. Ethanolic maceration of 900 g dried leaf powder produced 87.0 g crude ethanol extract, corresponding to a yield of 9.67%. Subsequent liquid–liquid partitioning produced n-hexane, ethyl acetate, and aqueous fractions, with the aqueous fraction representing the largest yield and the n-hexane fraction providing the targeted lipophilic fraction for GC–MS analysis.

GC–MS analysis of the selected nonpolar fraction resulted in the tentative annotation of 11 compounds, representing 94.44% of the total normalized peak area. The major tentatively annotated constituents were palmitic acid, phytol, squalene, and neophytadiene. Class-based interpretation showed that the selected fraction was dominated by diterpene-related compounds, fatty acids, and triterpene hydrocarbons, supporting the interpretation that the fractionation procedure enriched lipophilic plant constituents.

The reliability of the GC–MS fingerprint was supported by NIST 17 library matching, retention-index comparison using a C8–C40 n-alkane series, diagnostic ion evaluation, solvent blank analysis, and triplicate injections. All tentatively annotated compounds showed match scores of 90–98%, retention-index differences of 4–8 units, and RSD values of 1.86–3.27%, indicating acceptable spectral agreement, chromatographic support, and injection repeatability. Nevertheless, the compound assignments remain tentative because authentic reference standards were not used for final confirmation, and the reported area percentages should be interpreted as normalized GC–MS signal contributions rather than absolute concentrations.

From a green chemistry perspective, the present workflow showed only partial alignment with green extraction principles through the use of ethanol as the primary extraction solvent, natural sunlight drying, solvent recovery, and recirculating chiller-assisted evaporation. However, the continued use of n-hexane for nonpolar enrichment and chloroform for GC–MS sample preparation means that the overall procedure should not be described as a fully green extraction method. Therefore, the main contribution of this study is not the development of a completed green extraction protocol, but the generation of a transparent baseline nonpolar chemical fingerprint that can support future solvent-replacement and greener extraction optimization studies.

Future studies should compare this conventional n-hexane-based fractionation with safer and more sustainable systems, such as ethanol-water mixtures, bio-based solvents, ultrasound-assisted extraction, or microwave-assisted extraction. Further work should also include quantitative greenness assessment, including solvent consumption, solvent recovery, waste generation, energy demand, extraction efficiency, and analytical performance. Direct bioactivity assays and authentic-standard-based quantification are also needed before the detected lipophilic constituents can be linked to functional properties or proposed as confirmed chemical markers for quality control of *S. polyanthum* leaves.

## Figures and Tables

**Table 1:** Materials, reagents, and instruments used in the study.

Category	Item	Specification	Supplier/Manufacturer	Function in This Study
Plant material	*Syzygium polyanthum* leaves	Collected from Paniki Bawah, Mapanget, Manado	-	Botanical sample
Voucher specimen	*S. polyanthum*	SP-Mapanget-0623	-	Taxonomic traceability
Primary extraction solvent	Ethanol	Analytical grade (AR), 96%	Merck, Darmstadt, Germany	Maceration solvent
Aqueous phase	Distilled water	Distilled, warmed to 50 °C	Laboratory produced (Milli-Q^®^ system, MilliporeSigma, Burlington, MA, USA)	Suspension before partitioning
Nonpolar solvent	n-hexane	HPLC/GC grade, ≥99%	Merck, Darmstadt, Germany	Nonpolar partitioning and column elution
Semi-polar solvent	Ethyl acetate	Analytical grade (AR), ≥99.5%	Merck, Darmstadt, Germany	Semi-polar partitioning and column elution
GC–MS solvent	Chloroform	GC grade, ≥99.8%	Merck, Darmstadt, Germany	Sample dissolution before injection
RI standard	C8–C40 n-alkane standard mixture	40 mg/L each in hexane	Sigma-Aldrich, St. Louis, MO, USA	Retention-index calculation
Stationary phase	Silica gel 60	0.063–0.200 mm (70–230 mesh)	Merck, Darmstadt, Germany	Column chromatography
Sample adsorbent	Silica gel 60 GF254	10 g	Merck, Darmstadt, Germany	Dry loading of n-hexane fraction
TLC plate	Silica gel TLC plate	GF254, aluminum-backed	Merck, Darmstadt, Germany	Fraction monitoring
Rotary evaporator	Buchi R-100	-	Buchi, Flawil, Switzerland	Solvent removal
Analytical balance	Ohaus	-	Ohaus, Parsippany, NJ, USA	Sample weighing
Oven	Memmert	-	Memmert, Schwabach, Germany	Drying step when required
GC–MS system	Shimadzu GCMS-QP2010 Ultra	-	Shimadzu, Kyoto, Japan	Chemical profiling
Acquisition software	GCMSsolution	Version 4.45	Shimadzu, Kyoto, Japan	GC–MS data acquisition
Post-processing software	Agilent MassHunter Unknowns Analysis	Version B.10.1	Agilent Technologies, Santa Clara, CA, USA	Library-search support and data processing
Spectral library	NIST 17	National Institute of Standards and Technology	NIST, Gaithersburg, MD, USA	Compound annotation

**Table 2:** GC–MS operating conditions for analysis of the selected nonpolar fraction of *Syzygium polyanthum* leaves.

Parameter	Condition
Laboratory	Narkobafopol, Forensic Chemistry Division
Instrument	Shimadzu GCMS-QP2010 Ultra
Acquisition software	GCMSsolution Ver. 4.45
Post-processing software	Agilent MassHunter Unknowns Analysis
Library	NIST 17
Column	Agilent 19091S-433 HP-5MS
Column dimension	30 m × 0.25 mm × 0.25 μm
Injection solvent	Chloroform
Injection volume	1.0 μL
Injection mode	Split
Split ratio	1:50
Injector temperature	250 °C
Carrier gas	Helium, 99.999% purity
Carrier gas flow rate	1.0 mL/min, constant flow
Initial oven temperature	50 °C
Oven ramp rate	10 °C/min
Final oven temperature	280 °C
Final hold time	10 min
Ionization mode	Electron impact
Electron energy	70 eV
Ion source temperature	230 °C
Interface temperature	280 °C
Mass scan range	*m*/*z* 35–500
Solvent delay	4.0 min
Blank injection	Pure chloroform solvent blank
Replicate injection	Triplicate injections
Compound identification	Spectral library matching and retention index comparison
Identification status	Tentative, pending verification with authentic standards

**Table 3:** Green chemistry relevance and limitations of the experimental workflow.

Step	Solvent or Process Used	Green Chemistry Relevance	Limitation
Drying	Natural sunlight drying	Reduces external energy demand	Drying condition may vary with weather
Primary extraction	96% ethanol maceration	Ethanol is more acceptable than many petroleum-derived or chlorinated solvents	Maceration is time-consuming and solvent-intensive
Solvent recovery	Rotary evaporation	Approximately 75% recovery of ethanol and n-hexane	Solvent loss still occurs
Cooling system	Recirculating chiller	Reduces water waste during evaporation	Energy consumption was not quantitatively measured
Redissolution	Warm distilled water	Water is environmentally preferable and safe	Limited ability to dissolve nonpolar constituents
Nonpolar partitioning	n-hexane	Enriches lipophilic compounds for GC–MS fingerprinting	Petroleum-derived solvent; not preferred in green chemistry
Semi-polar partitioning	Ethyl acetate	Useful for polarity-based fractionation	Still an organic solvent and requires recovery or reduction
GC–MS preparation	Chloroform	Efficient dissolution of nonpolar analytical sample	Hazardous chlorinated solvent
Chemical profiling	GC–MS, NIST library matching, RI calculation	Supports detailed profiling of GC–MS-detectable volatile constituents	Compound identification remains tentative without authentic standards
Future improvement	Ethanol-water mixtures, bio-based solvents, ultrasound-assisted extraction, microwave-assisted extraction	Supports greener method development	Requires comparative optimization and validation

**Table 4:** Extraction and fractionation yields of *Syzygium polyanthum* leaves.

Sample or Fraction	Weight Obtained	Yield Basis	Yield (%)
Crude ethanol extract	87.0 g	Dried leaf powder, 900 g	9.67
n-Hexane fraction	5.2 g	Crude ethanol extract, 87.0 g	5.98
Ethyl acetate fraction	18.4 g	Crude ethanol extract, 87.0 g	21.15
Aqueous fraction	63.4 g	Crude ethanol extract, 87.0 g	72.87

**Table 5:** Tentatively annotated compounds in the selected nonpolar fraction of *Syzygium polyanthum* leaves by GC–MS.

No.	RT (min)	Tentative Compound	Chemical Class	Area % (Mean ± SD)	Identification Status
1	8.42	α-Pinene	Monoterpene hydrocarbon	2.15 ± 0.04	Tentative, RI-supported
2	11.15	L-Linalool	Monoterpene alcohol	1.84 ± 0.05	Tentative, RI-supported
3	14.21	β-Caryophyllene	Sesquiterpene hydrocarbon	4.38 ± 0.12	Tentative, RI-supported
4	16.55	Neophytadiene	Diterpene-related compound	12.45 ± 0.31	Tentative, RI-supported
5	18.12	Palmitic acid	Fatty acid	24.12 ± 0.48	Tentative, RI-supported
6	20.45	Phytol	Diterpene-related compound	18.67 ± 0.52	Tentative, RI-supported
7	22.18	Stearic acid	Fatty acid	5.82 ± 0.14	Tentative, RI-supported
8	25.64	Squalene	Triterpene hydrocarbon	15.34 ± 0.38	Tentative, RI-supported
9	28.9	α-Tocopherol	Tocopherol	3.10 ± 0.09	Tentative, RI-supported
10	31.15	Stigmasterol	Phytosterol	2.45 ± 0.08	Tentative, RI-supported
11	33.45	β-Sitosterol	Phytosterol	4.12 ± 0.11	Tentative, RI-supported
		Total annotated area		94.44	

**Table 6:** Chemical-class distribution of Tentatively annotated constituents in the selected nonpolar fraction.

Chemical Class	Compounds	Count	Total Area (%)
Monoterpene hydrocarbons	α-Pinene	1	2.15
Monoterpene alcohols	L-Linalool	1	1.84
Sesquiterpene hydrocarbons	β-Caryophyllene	1	4.38
Diterpene-related compounds	Neophytadiene; Phytol	2	31.12
Fatty acids	Palmitic acid; Stearic acid	2	29.94
Triterpene hydrocarbons	Squalene	1	15.34
Tocopherols	α-Tocopherol	1	3.1
Phytosterols	Stigmasterol; β-Sitosterol	2	6.57
Total annotated area	-	11	94.44

**Table 7:** Comparison between the present study and previous *Syzygium polyanthum* fractionation studies.

Aspect	Previous Syabana et al. Study	Present Study
Main purpose	Bioactivity-oriented fractionation and identification of antidiabetic-related candidates	Baseline GC–MS fingerprinting of lipophilic constituents
Extraction/fractionation approach	Solvent-gradient fractionation using n-hexane, acetone, and water	Ethanol extraction, liquid–liquid partitioning, silica gel chromatography, and TLC-guided selection
Number of fractions	42 fractions	Selected combined nonpolar fraction from vials 15–28
Main analytical focus	Bioactivity-related chemical profiling	GC–MS profiling supported by RI, match score, diagnostic ions, blank analysis, and triplicate injection
Main chemical emphasis	Polar or semi-polar bioactivity-related constituents	GC–MS-detectable volatile lipophilic constituents
Main compounds emphasized	Antidiabetic-related marker candidates	Palmitic acid, phytol, squalene, neophytadiene, terpenoids, tocopherol, and phytosterols
Geographic/source emphasis	Not focused on North Sulawesi regional fingerprinting	Leaves collected from Paniki Bawah, Mapanget District, Manado, North Sulawesi
Main contribution	Bioactivity-oriented fractionation of *S. polyanthum* leaves	Region-specific, RI-supported nonpolar GC–MS fingerprint as a baseline for future greener extraction optimization

## Data Availability

The original contributions presented in this study are included in the article/Supplementary Materials. Further inquiries can be directed to the corresponding author.

## Supplementary Materials

The following supporting information can be downloaded at: https://www.mdpi.com/article/10.3390/molecules31111932/s1, Table S1: Detailed GC–MS annotation parameters of tentatively annotated compounds in the selected nonpolar fraction of *Syzygium polyanthum* leaves, including retention time, molecular formula, molecular weight, experimental retention index, reference retention index, ΔRI, NIST 17 match score, diagnostic ions, normalized peak area, RSD, blank assessment, and identification status.
