# Supplementary material for: Solvent-Dependent GC–MS Fingerprinting of Lipophilic Constituents in Syzygium polyanthum Leaves: A Baseline Study for Future Greener Extraction Optimization
Source: Molecules. 2026 Jun 3;31(11):1932. doi: 10.3390/molecules31111932 (PMC13258493; doi:10.3390/molecules31111932)
Supplement: Supplementary file 1 [file molecules-31-01932-s001.zip › molecules-4251208-supplementary.pdf]

Supplementary Material Table S1

Solvent-Dependent GC–MS Fingerprinting of Lipophilic Constituents in *Syzygium polyanthum* Leaves: A Baseline Study for Future Greener Extraction Optimization

Frangky J. Paat and Sanriomi Sintaro

No.	RT (min)	Tentative compound	Formula	MW	Exp. RI	Ref. RI	ΔRI	Match score (%)	Diagnostic ions (m/z)	Area % (mean ± SD)	RSD (%)	Identification status
1	8.42	α-Pinene	$C_{10}H_{16}$	136	935	939	4	94	93, 91, 77	2.15 ± 0.04	1.86	Tentative, RI-supported
2	11.15	L-Linalool	$C_{10}H_{18}O$	154	1102	1097	5	91	71, 93, 121	1.84 ± 0.05	2.72	Tentative, RI-supported
3	14.21	β-Caryophyllene	$C_{15}H_{24}$	204	1424	1419	5	96	93, 133, 161	4.38 ± 0.12	2.74	Tentative, RI-supported
4	16.55	Neophytadiene	$C_{20}H_{38}$	278	1836	1830	6	95	68, 82, 95	12.45 ± 0.31	2.49	Tentative, RI-supported
5	18.12	Palmitic acid	$C_{16}H_{32}O_2$	256	1968	1960	8	98	73, 129, 256	24.12 ± 0.48	1.99	Tentative, RI-supported
6	20.45	Phytol	$C_{20}H_{40}O$	296	2114	2110	4	96	71, 81, 123	18.67 ± 0.52	2.78	Tentative, RI-supported
7	22.18	Stearic acid	$C_{18}H_{36}O_2$	284	2172	2167	5	97	73, 129, 284	5.82 ± 0.14	2.41	Tentative, RI-supported
8	25.64	Squalene	$C_{30}H_{50}$	410	2824	2820	4	98	69, 81, 410	15.34 ± 0.38	2.48	Tentative, RI-supported
9	28.9	α-Tocopherol	$C_{29}H_{50}O_2$	430	3155	3148	7	92	165, 205, 430	3.10 ± 0.09	2.9	Tentative, RI-supported
10	31.15	Stigmasterol	$C_{29}H_{48}O$	412	3295	3287	8	90	55, 83, 412	2.45 ± 0.08	3.27	Tentative, RI-supported
11	33.45	β-Sitosterol	$C_{29}H_{50}O$	414	3358	3351	7	90	414, 396, 213	4.12 ± 0.11	2.67	Tentative, RI-supported
